# Teacher Self-Efficacy and Mental Health—Their Intricate Relation to Professional Resources and Attitudes in an Established Manual-Based Psychological Group Program

**DOI:** 10.3389/fpsyt.2021.510183

**Published:** 2021-05-28

**Authors:** Sophia von Muenchhausen, Matthias Braeunig, Ruth Pfeifer, Anja S. Göritz, Joachim Bauer, Claas Lahmann, Alexander Wuensch

**Affiliations:** ^1^Department of Psychosomatic Medicine and Psychotherapy, Faculty of Medicine, Medical Center—University of Freiburg, Freiburg, Germany; ^2^Occupational and Consumer Psychology, University of Freiburg, Freiburg, Germany; ^3^International Psychoanalytic University, Berlin, Germany

**Keywords:** teachers, self-efficacy, mental health, teacher health, prevention, manual-based intervention, work-related behavior and experience patterns

## Abstract

**Introduction:** Teaching is considered a mentally challenging occupation. Teacher self-efficacy is a personal resource which buffers the experience of stress and may be important in maintaining mental health. The preventive intervention “Manual-Based Psychological Group Program for Teachers” (MBPGPT) was applied and evaluated state-wide to improve the mental health of teachers. This study aims to investigate the intricate relation between teacher self-efficacy and mental health and their changes in the course of the intervention.

**Method:** Using a single-group pre-/post-design, the relation between teacher self-efficacy and mental health was investigated in 742 teachers. Pre- and post-changes in teacher self-efficacy and their interaction with mental health were examined in a subsample of 171 teachers, who met the conservative inclusion criteria. In ancillary analyses, correlations with underlying changes in work-related behavior and experience patterns were analyzed to better understand the intricate link between teacher self-efficacy and mental health.

**Results:** Teacher self-efficacy showed a significant, moderate correlation with mental health. Self-efficacy was moderately higher after the intervention than before the intervention, but independent of changes in mental health. Teacher self-efficacy was related to work-related psychological resistance and positive emotions. An increase in teacher self-efficacy was accompanied by an improvement in life satisfaction and distancing ability. A decrease in teacher self-efficacy went hand in hand with reduced experience of social support.

**Discussion:** This study confirmed teacher self-efficacy as an important, reliable resource and its correlation with psychological resistance. The absence of a control group limits what causal conclusions can be drawn from the study. Nevertheless, self-efficacy seems to be a worthwhile goal of preventive interventions for teachers and should be promoted due to its wide-ranging implications. Suggestions for further studies and interventions are made.

## Introduction

Teaching is a profession characterized by daily emotional interactions and is accompanied by a variety of demands regarding cultural, societal, and social aspects. Teachers are influenced by different role expectations, such as being an educator, mediator, and manager. These roles include coping with a variety of tasks, challenges, and multifactorial demands (1, 2). Consequently, teaching is regarded as a profession with high psycho-emotional stress (2–5). Teachers often experience intense work-related stress which, if experienced chronically, may result in burnout (6). Resignation and exhaustion are higher among teachers compared with other highly psycho-socially demanding professions (7). Ten to Fifteen years ago, ~30% of German teachers reported burnout symptoms (8–11). In 2005, 60% of German teachers retired early, and among these, 52% retired prematurely due to mental illnesses such as recidivated depression and burnout (12). These rates have remained high: From 2011 to 2013, 9–13% of teachers in the German state of Baden–Württemberg retired early due to health issues, and among these, 55–57% retired prematurely because of mental illnesses and behavioral disorders (13).

The socio-psychological stress in teaching results from structural factors such as time pressure, workload, role conflict, and increasing class sizes (6, 14, 15). Additionally, due to the daily interpersonal interactions, negative experiences in the relationship with students, parents, and colleagues are risk factors (3, 16, 17). Destructive student behavior is one of the most important determinants of teacher health (3, 17, 18). However, positive feedback from students, parents, and colleagues counts as an important resource to prevent mental and physical strain (19). There is a negative correlation between perceived social support and mental health as well as with the number of sick days (20). Moreover, experienced social support decreases the taxing effect of destructive student behavior (ibid.). Thus, the quality of the relationship between teacher and students influences teacher health: A successful teacher–student relationship may reduce the straining effect of destructive student behavior and, thus, mediate the negative relationship between destructive student behavior and teacher health (21). Additionally, a successful teacher–student relationship boosts student performance. Teachers who act in a person-centered and relationship-oriented way create an open learning situation in which students show less defiant behavior and higher commitment, respect, and performance (22).

The Manual-Based Psychological Group Program for Teachers (MBGPT) (23) aims at fostering teacher's competency in relationship-building to maintain their long-term mental and physical health (23, 24). The intervention consists of five modules that focus on how negative and positive relationships affect teacher health: (1) basic knowledge of stress physiology and effects on health parameters, (2) mental attitudes focusing on authenticity and identification, (3) competence in handling relationships with students, (4) competence in handling relationships with parents, and (5) collegiality and social support among the staff (23). A main feature of the intervention is Balint group work [e.g., (25, 26)]. Balint group work fosters the transfer of the perspective of all people who are involved in a discussion of a specific situation; furthermore, it facilitates insight into defective relationship processes and enhances solution-oriented approaches (23). Significant improvements in mental health have been demonstrated in a RCT on teachers who participated in five of ten sessions of the MBPGPT (27, 28). However, for practical reasons the current program has been shortened to six consecutive sessions. There were two versions: Participants could choose between participating in either a compact version with the six sessions delivered over a day and a half or in a version that was stretched out over an extended time period with, e.g., one session a month.

In addition to improving mental health in general (27, 28), the baseline of the assessed health measures was the strongest predictor of health improvement (29). Consequently, teachers who suffered most benefited most from the intervention. Moreover, participating teachers changed work-related behavior and experience patterns (30) as measured by the AVEM inventory (“Arbeitsbezogene Verhaltens- und Erlebensmuster”) (31). Teachers whose mental health improved showed a reduced willingness to work until exhaustion, reduced perfectionism, and a reduced tendency for resignation in the face of failure. Moreover, those teachers increased their ability to distance themselves from occurrences in school, their inner calm and balance, and general life satisfaction (30).

The aim of this study was to investigate how the intervention impacted teachers' mental health by exploring correlations with and changes in teacher self-efficacy. General self-efficacy, as part of Bandura's (32) social cognitive theory, is a key element in self-regulated motivational and volitional goal orientation (33). Teacher self-efficacy describes a teacher's confidence in his or her capabilities to successfully carry out goal-oriented, occupation-related activities and to positively influence students' learning behavior (34). The construct of teacher self-efficacy can change depending on personal attributions and environmental circumstances (35). Also, as individual work-related tasks ask for different competencies, teacher self-efficacy can vary between different task areas and, thus, shows a high specificity (36).

Teacher self-efficacy is a personal resource (37). High teacher self-efficacy protects against occupational and health-related strains (38–40). It correlates positively with work satisfaction (34, 41, 42), engagement (35, 43), occupational commitment (44, 45), a proactive attitude, and school-related activities beyond the school environment (46). Moreover, teacher self-efficacy correlates negatively with stress and the burnout factors leading to reduced personal accomplishment, emotional exhaustion, and depersonalization (38, 46, 47). Along with the health benefits, teachers with high self-efficacy tend to be more efficient (48) and interact more effectively with their students (49). These teachers create an advantageous learning environment where destructive student behavior is reduced (39) and higher student performances may result (50).

Within the modules of the MBOGPT (15), teacher self-efficacy is not explicitly addressed. However, implicit and plausible connections between teacher self-efficacy and the group intervention could be identified. For example, the intervention supports teachers' confidence to actively access resources to feel competent, capable of acting, and goal-oriented. Moreover, the intervention aims at fostering teachers' capabilities for building successful relationships that provide social support, which in turn acts as an important source of teacher self-efficacy (51).

Specifically, we postulated four hypotheses: H1: There is a negative correlation between self-efficacy and mental health (correlational hypothesis). H2: As mental strain decreased pre- and post-intervention by a medium effect (27, 28) and as teacher self-efficacy is thought to protect against mental health impairments, teacher self-efficacy was expected to increase from pre- to post-intervention by a medium effect (growth hypothesis). H3: Along with the finding that teachers who suffered most benefited most from the intervention, baseline self-efficacy negatively predicts the pre-/post-change in teacher self-efficacy (regression hypothesis). H4: Teacher self-efficacy and mental health interact such that the pre-/post-change in mental health influences how teacher self-efficacy changes: Teachers whose mental health had improved post-intervention experienced a stronger growth in their self-efficacy post-intervention than teachers whose mental health did not change or worsened post-intervention (moderation hypothesis).

To better understand the relationship between teacher self-efficacy and mental health, we explored the relationships between teacher self-efficacy and work-related attitudes and behavior patterns as measured by the AVEM inventory (31) in two ancillary analyses. The first ancillary analysis examined how teachers with low, medium, and high self-efficacy differed on AVEM subscales or features before the intervention. The second ancillary analysis examined how and how much (in terms of effect size) particular work-related attitudes and behavior patterns changed as teacher self-efficacy improved or worsened from pre- to post-intervention.

## Materials and Methods

### Sample and Design

The data resulted from the preventive coaching offered to all school teachers in the state of Baden-Württemberg, Germany, by the State Ministry of Culture (Ministerium für Kultur, Jugend und Sport Baden-Württembergs). Teachers who registered for MBPGPT (23) participated in the intervention and its accompanying evaluation study. Recruitment and data collection took place from April 2016 to July 2019, comprising two consecutive school years (2017/2018 and 2018/2019). Within each of the 2 school years, the program ran from October to April. Every group consisted of up to 12 teachers, whereas school principals formed separate groups to prevent conflicts of interest and to respond to their different needs. Licensed psychotherapists with a psychological or medical background delivered the intervention. By means of the manual (23), the psychotherapists presented the theoretical input of each module and facilitated the Balint group work later on.

**Figure 1:**
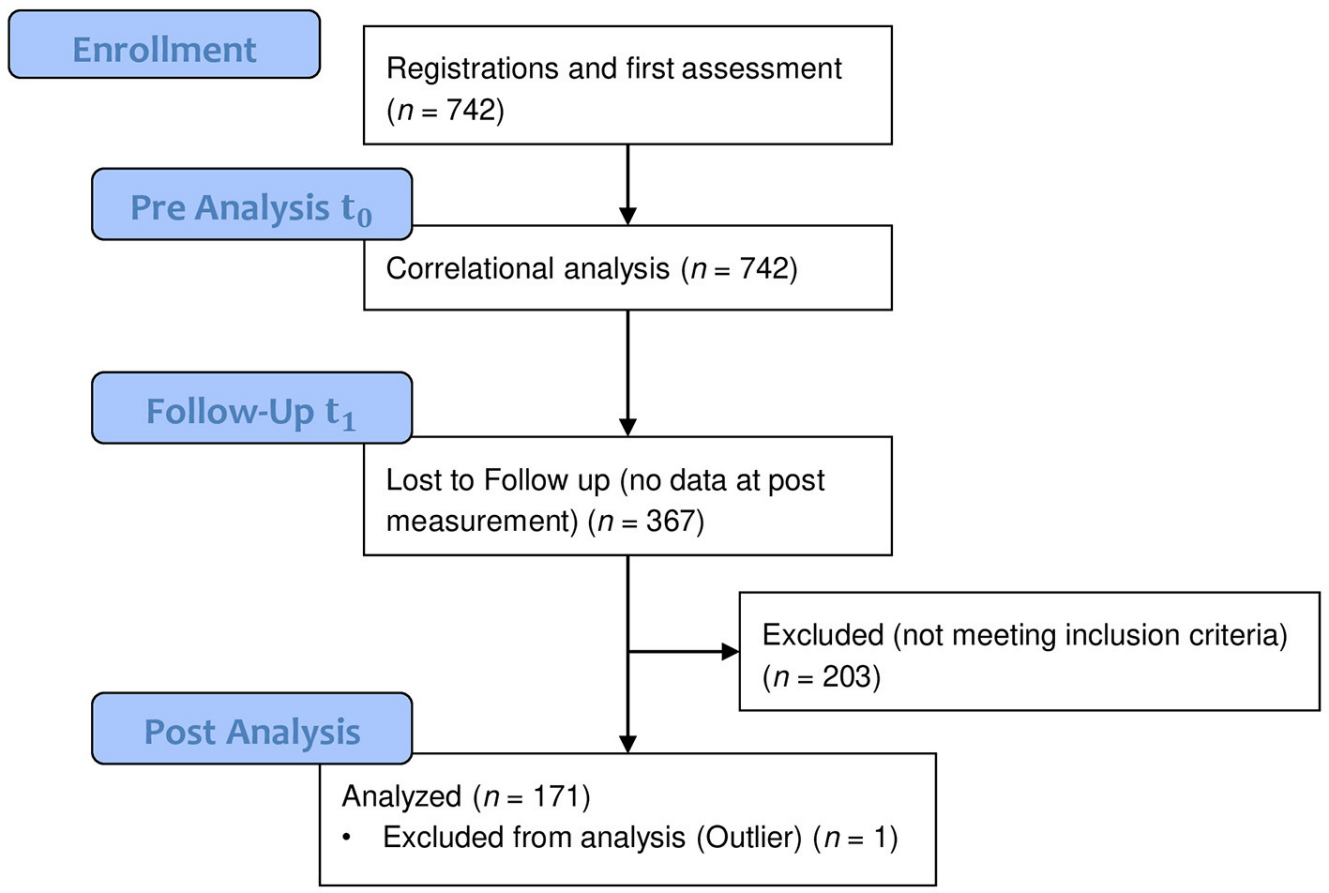
Phases of data assessment and analyses.

Teachers who registered for the intervention filled out an online questionnaire before the intervention to measure psychological factors at baseline (*t*_0_) and again 2 weeks after completing the intervention (*t*_1_). No control group existed as the intervention was offered as a preventive program for everybody. Teachers participated voluntarily. To remain anonymous, each teacher generated an individual code according to a given pattern. Across both school years, *N*_*t*__0_ = 742 registered for the intervention and answered the pre-intervention questionnaire. *N*_*t*__1_ = 375 participants remained who answered the post-questionnaire. Both questionnaires were matched by the teacher-generated code and their sex. Furthermore, other sociodemographics were examined with filters to ensure that all data were matched correctly. High standards for inclusion in the pre- and post-analyses were applied: participation in at least five out of six sessions or, alternatively, the full-day seminar plus submitted data from both pre- and post-questionnaire. A total of *n* = 172 teachers met the inclusion criteria for the pre- and post-analyses. For an overview of the phases of data assessment and analyses, see Figure 1.

The subgroup of the 172 teachers differed from the 742 teachers who had participated at *t*_0_: The subgroup was older and had a higher percentage of elementary school teachers and a higher percentage of part-time teachers. However, they were comparable in the fraction of principals and in their family status. Moreover, the key variables teacher self-efficacy (TSE), *t*_TSE_(740) = −0.72, *p* = 0.4, and GHQ-12 (general mental health) did not differ, *t*_GHQ−12_(740) = 0.63, *p* = 0.52.

Within the subsample of 172 teachers, 142 were women (82.6%). The age category “55 years and older” was most frequent with 48 teachers (27.9%), followed by the age category “45–49 years” with 43 teachers (25%). The age category “under 35 years” was least frequent with 13 teachers (7.6%). The teachers had taught 16.5 years on average. The majority (141) was in a relationship (82%), and 116 teachers were married (67.4%). Most teachers (52) taught in elementary school (34.9%), 34 in professional school (19.8%), 29 in junior high school (16.9%), 26 in high school (15.1%), 17 in special needs education school (9.9%), 5 in community school (2.9%), and 1 in school kindergarten (0.6%). Nearly half of the teachers (82) were employed full time (47.7%), about one third (53) were employed part-time at 50–75% (31.4%), 24 taught part-time at >75% (14%), and 12 taught part time at <50%. Among the participants, 21 were school principals (12.2%).

### Measures

Data were collected on teacher self-efficacy, general mental health, work-related attitudes, behavior patterns, and sociodemographics.

### Teacher Self-Efficacy

TSE was measured with LehrWirk (54). This inventory consists of 10 items that are answered on a four-point scale (1 = does not apply to 4 = does apply very much) and capture teacher self-efficacy as a general, one-dimensional construct. Sample items are as follows: “I am certain that I can build a good relationship even with the most problematic students” and “Even if my lessons are disturbed, I am certain to maintain inner calmness.” The inventory is based on four competency demands within the teaching profession: social interaction, expectations on performances, handling of emotions, and innovation. Internal consistency varies between α = 0.76 and 0.82 (54). A 3-year retest reliability varies between *r*_tt_ = 0.65 and 0.61 (54). In the present study, internal consistencies were α = 0.79 at *t*_0_ and α = 0.81 at *t*_1_.

### General Mental Health

The German version of the General Health Questionnaire (GHQ-12) (55) was administered. GHQ-12 is a screening instrument and measures the current mental health status by one general factor. It shows a reliability of α = 0.89 (56). WHO studies confirmed the validity of the GHQ-12 as a screening instrument (56, 57). GHQ-12 allows dichotomizing the score at a cutoff value ≥4: A GHQ-12 score ≥4 indicates a mental health status at risk, while a score < 4 indicates good mental health status (57).

### Work-Related Behaviors and Experience Patterns

Work-related behavior and experience patterns (AVEM) were measured using the AVEM-44 scale (31). This short version of the AVEM includes 11 subscales with four items each that are answered on a five-point scale (1 = applies completely, 5 = applies not at all). The subscales or AVEM features are as follows: (1) subjective importance of work (BA), (2) professional ambition (BE), (3) willingness to work to exhaustion (VB), (4) striving for perfection (PS), (5) distancing ability (DF), (6) tendency for resignation in the face of failure (RT), (7) proactive problem-solving (OP), (8) inner calm and balance (IR), (9) experience of success at work (EE), (10) general life satisfaction (LZ), and (11) experience of social support (SU). The first four features represent the resource work commitment, 5–8 indicate the resource psychological resistance, and the last three features capture the resource emotions. Normed scales with stanine values (*M* = 5, SD = 2) are available for German teachers. AVEM-44 shows good internal consistency for all 11 subscales (α = 0.76–0.83). The stability is lower due to the subscales' variability regarding time and context. Therefore, AVEM-44 is a reliable measure as well as sensitive toward changes.

### Data Analyses

Analyses were performed using IBM SPSS Statistics 25 and PROCESS macro 3.3 (53). Prior to the analyses, the data were investigated descriptively to examine the statistical assumptions and identify potential outliers. The distributions of the data within the variables were non-normal, except for teacher self-efficacy at *t*_0_. Therefore, mostly nonparametric tests were used [cf. (58)]. One outlier was identified whose self-efficacy worsened dramatically. A screening of the other variables revealed that this person also reported worsening of her already at-risk mental health status as well as her work-related attitudes, and behavioral patterns worsened. Because the group program is designed as a preventive intervention and has been proven to foster teacher's health, we concluded that the explanation for the person's reports lies elsewhere. Therefore, this case was excluded from the analyses, and the pre-/post-sample reduced to *n* = 171.

To test the correlational hypothesis H1, we correlated GHQ-12 and teacher self-efficacy at both measurement points. To test the growth hypothesis H2, Wilcoxon signed-rank tests with dependent samples were conducted to test pre- and post-changes in teacher self-efficacy. To test the regression hypothesis H3, we first calculated an indicator of the pre-/post-change in teacher self-efficacy (δTSE), which was the difference between measurement *t*_1_ and *t*_0_ (δTSE = TSE*t*_1_ – TSE_t0_). The higher the value of δTSE, the higher the improvement in self-efficacy throughout the intervention. Next, we regressed δTSE on self-efficacy at *t*_0_ (TSE_t0_). To test the moderation hypothesis H4, which postulates that a change in mental health has an effect on the relationship between teacher self-efficacy *t*_0_ and pre-/post-change in teacher self-efficacy, we first calculated an indicator of the change in mental health (δGHQ), which was the difference between GHQ-12 pre- and post-intervention (δGHQ = GHQ-12*t*_1_ – GHQ-12*t*_0_). Because lower values of GHQ-12 indicate a better mental health status, δGHQ was negative if a participant improved his or her mental health status throughout the intervention. With PROCESS (53), the multiplicative term (δGHQ × TSE_t0_) was entered to test the moderating effect of the change in mental health. To ensure homoscedasticity, PROCESS uses heteroskedastic robust standard deviations (Huber–White). To investigate the form of the moderation, a simple slope test was performed, again using the PROCESS macro 3.0. We analyzed the conditional effects via simple slopes for low (16th percentile), medium (50th percentile), and high (84th percentile) levels of the moderator (δGHQ).

For the ancillary analysis, we performed a discriminant analysis based on all registered teachers before the intervention (*t*_0_) *n* = 742.

In the ancillary analyses, we first grouped the 742 teachers pre-intervention (*t*_0_) according to their self-efficacy at baseline (TSE_t0_): very low (TSE_t0_ ≤ *M* – 2 SD), low (TSE_t0_ ≤ *M* – 1 SD), medium (*M* – 1 SD < TSE_t0_ < *M* + 1 SD), high (TSE_t0_ ≥ *M* + 1 SD), and very high (TSE_t0_ ≥ *M* + 2 SD). Based on these levels of self-efficacy at baseline, we calculated means and standard deviations and estimated 95% confidence intervals for each group on each AVEM subscale. Second, we grouped the participants with both pre- and post-data (*n* = 171) according to whether their self-efficacy improved (dTSE ≥ *M* + 1 SD), remained constant (*M* – 1 SD ≤ dTSE ≤ *M* + 1 SD), or deteriorated (dTSE ≤ *M* – 1 SD). Again, based on these levels of change in teacher self-efficacy pre- and post-intervention, we calculated means and standard deviations for each group on each AVEM subscale, separately for *t*_0_ and *t*_1_. Next, we compared the means of *t*_0_ and *t*_1_ and calculated the effect size *d* for each AVEM subscale to examine how the scores on the subscales changed for each of the three groups.

## Results

### Correlations and Pre- and Post-changes in Teacher Self-Efficacy

First, at both time points, the higher the teachers' self-efficacy, the better their general mental health (GHQ-12) (*t*_0_: *r*_s_ = −0.285, *p* < 0.001, *t*_1_: *r*_s_ = −0.270, and *p* < 0.001). This supports the correlation hypothesis H1. Second, teacher self-efficacy was higher at *t*_1_ (median = 30) than at *t*_0_ (median = 28), *z* = 5.81, *p* < 0.001, and *r* = 0.31. These results support the growth hypothesis H2. The change was medium in size (59). Third, in the regression analysis, teacher self-efficacy at *t*_0_ predicted the pre-/post-change in teacher self-efficacy (*b* = −0.253, 95% CI = −0.37, −0.132, *t* = −4.11, and *p* < 0.001), thus supporting the regression hypothesis H3 (Table 1). Fourth, the moderation hypothesis H4 was not supported, as the interaction between teacher self-efficacy at baseline and pre-/post-change in mental health was not significant (*b* = 0.01, 95% CI = −0.03, 0.05, *t* = 0.45, and *p* = 0.64; Table 1). The simple slope analysis of the conditional effect for low (16th percentile = −5.48), medium (50th percentile = −1), and high (84th percentile = 1) levels of the moderator (i.e., pre-/post-change in mental health) revealed that as teacher self-efficacy was already fairly high at *t*_0_ (*M* + 1 SD, TSE_t0_ = 32), it remained the same at *t*_1_, independent of how the mental health status had changed before and after (not shown).

**Table 1:** Estimates of the main effect of teacher self-efficacy at baseline measure (TSE_t0_) and the interaction effect of pre-/post-change in mental health (dGHQ) on pre-/post-change in teacher self-efficacy.

**Variables**	**Pre-/post-change in teacher self-efficacy**	
	***b* (95% CI)**	**SE *b***
**Step 1: simple regression analysis**		
Teacher self-efficacy baseline (TSE_t0_)	−0.25^**^ (−0.37, −0.13)	0.06
	*R*^2^ *=* 0.091	
**Step 2: moderation analysis**		
Teacher self-efficacy baseline (TSE_t0_)	−0.23^**^ (−0.35, −0.10)	0.06
Pre-/post-change in mental health status (dGHQ)	−0.34 (−1.57, 0.89)	0.62
TSE_t0_ × dGHQ	0.01 (−0.03, 0.05)	0.02
	*R*^2^ = 0.096	

*TSE_t0_, teacher self-efficacy at baseline measurement; dGHQ, pre-/post-change in mental health status*.

** *p < 0.001*.

### Ancillary Analyses of Self-Efficacy and AVEM Subscales or Features

**Figure 2:**
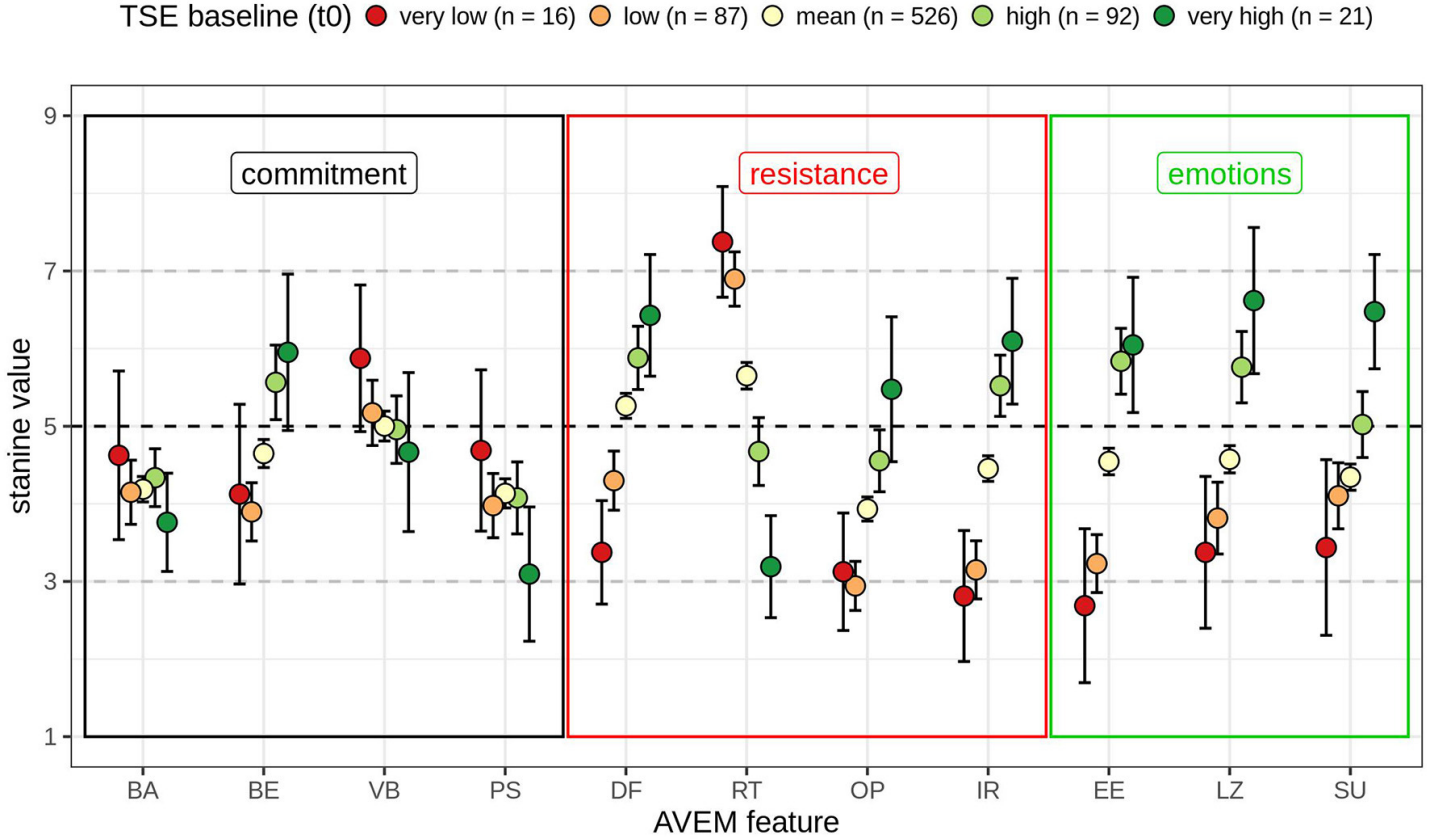
Teacher self-efficacy at *t*_0_ (*N* = 742; very low: TSE_t0_ ≤ *M* – 2 SD; low: *M* – 2 SD < TSE_t0_ ≤ *M* – 1 SD; mean: *M* – 1 SD < TSE_t0_< *M* + 1 SD; high: *M* < TSE_t0_ ≤ *M* + 1 SD; very high: *M* + 2 SD ≤ TSE_t0_) with mean stanine values (*M* = 5, SD = 2) and 95% confidence interval by AVEM subscale or feature (BA, subjective importance of work; BE, professional ambition; VB, willingness to work to exhaustion; PS, striving for perfection; DF, distancing ability; RT, resignation tendency toward failure; OP, proactive problem-solving; IR, inner calm and balance; EE, experience of success at work; LZ, general life satisfaction; SU, experience of social support). The AVEM subscales or features are grouped within their corresponding resources: commitment, work commitment (black); resistance, psychological resistance factors (red), and emotions (green).

First, the discrimination analysis with all 742 registered teachers showed how teachers differed by AVEM subscale or feature, as teachers were divided into five groups based on their baseline score of self-efficacy (Figure 2).

Teachers with (1) *very low teacher self-efficacy* (*n* = 16) had average scores on the AVEM features that make up the resource work commitment. Only subjective importance of work (BE) was low, though still within the normal range (*M*_BE_ = 4.13, 95% CI = 2.97, 5.28). Scores on AVEM features that represent the resource psychological resilience were mostly outside the normal range. These teachers also had low distancing ability (DF, *M*_DF_ = 3.38, 95% CI = 2.71, 4.04), a high tendency for resignation (RT, *M*_RT_ = 7.38, 95% CI = 6.66, 8.09), low proactive problem-solving (OP, *M*_OP_ = 3.13, 95% CI = 2.37, 3.88), and low inner calm and balance (IR, *M*_IR_ = 2.81, 95% CI = 1.97, 3.66). Scores on AVEM features that represent the resource emotions were rather low, and all scores were below the mean (experience of success at work, EE, *M*_EE_ = 2.69, 95% CI = 1.70, 3.68; general life satisfaction, LZ, *M*_LZ_ = 3.38, 95% CI = 2.4, 4.35; experience of social support, SU, *M*_SU_ = 3.44, 95% CI = 2.31, 4.57). Teachers with (2) *low teacher self-efficacy* (*n* = 87) scored within the normal range on the AVEM features that indicate work commitment. By contrast, scores on AVEM features that represent psychological resilience were mostly outside the normal range, but less extreme than in the group with very low self-efficacy. Only distancing ability (DF) was fairly low, but within the normal range (*M*_DF_ = 4.3, 95% CI = 3.92, 4.68). Scores on the AVEM features that indicate emotions were also lower than the mean, but only experience of success at work was outside the normal range (*M*_EE_ = 3.23, 95% CI = 2.86, 3.6). Teachers with (3) *medium teacher self-efficacy* (*n* = 526) scored within the normal range on all AVEM subscales. Teachers with (4) *high teacher self-efficacy* (*n* = 92), just like the group with medium self-efficacy, had average scores on all AVEM subscales. However, as the confidence intervals indicate, some AVEM subscales differed as compared with the group with medium self-efficacy. On the features that represent the resource work commitment, teachers with high teacher self-efficacy had more professional ambition (BE, *M*_BE_ = 5.57, 95% CI = 5.08, 6.05). On the features that reflect psychological resilience, these teachers had higher distancing ability (DF, *M*_DF_ = 5.88, 95% CI = 5.74, 6.29), lower tendency for resignation (RT, *M*_RT_ = 4.67, 95% CI = 4.24, 5.11), higher proactive problem-solving (OP, *M*_OP_ = 4.55, 95% CI = 4.16, 4.95), and higher inner calm and balance (IR, *M*_IR_ = 5.52, 95% CI = 5.13, 5.92). On the features that represent the resource emotions, they reported to experience more success at work (EE, *M*_EE_ = 5.84, 95% CI = 5.41, 6.26), higher general life satisfaction (LZ, *M*_LZ_ = 5.76, 95% CI = 5.3, 6.22), and higher experience of social support (SU, *M*_SU_ = 5.02, 95% CI = 4.6, 5.45). Teachers with (5) *very high teacher self-efficacy* showed scores similar to teachers with high teacher self-efficacy, but the confidence intervals on some subscales indicate values out of the normal range. Among the features that represent work commitment, professional ambition (BE) was rather high, though still within the normal range (*M*_BE_ = 5.95, 95% CI = 4.94, 6.96). Striving for perfection (PS) was rather low with confidence intervals indicating scores outside the normal range (*M*_PS_ = 3.1, 95% CI = 2.23, 3.96). Among the features that reflect psychological resilience, these teachers showed rather high distancing ability (DF, *M*_DF_ = 6.43, 95% CI = 5.64, 7.21) and rather low tendency for resignation (RT, *M*_RT_ = 3.19, 95% CI = 2.53, 3.85). On the features that represent emotions, these teachers reported high life satisfaction (LZ, *M*_LZ_ = 6.62, 95% CI = 5.68, 7.56) and high experience of social support (SU, *M*_SU_ = 6.48, 95% CI = 5.74, 7.21).

**Figure 3:**
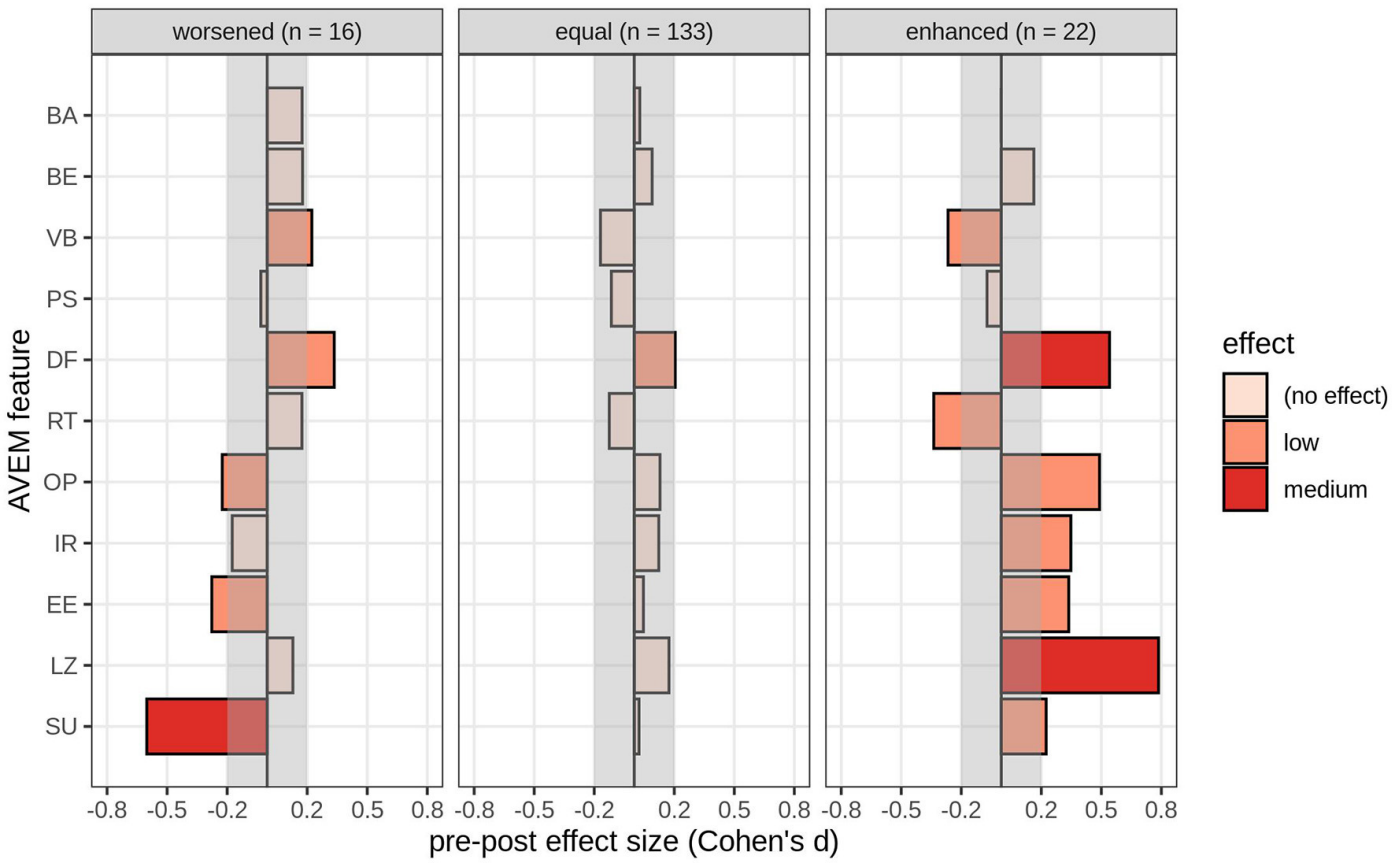
Effect sizes pre- and post-intervention on each AVEM subscale or feature (BA, subjective importance of work; BE, professional ambition; VB, willingness to work to exhaustion; PS, striving for perfection; DF, distancing ability; RT, resignation tendency toward failure; OP, proactive problem-solving; IR, inner calm and balance; EE, experience of success at work; LZ, general life satisfaction; SU, experience of social support), grouped by how teacher self-efficacy changed pre- and post-intervention (δTSE; worsened: δTSE ≤ *M* – 1 SD; equal: *M* – 1 SD < δTSE < *M* + 1 SD; enhanced: δTSE ≥ *M* + 1 SD).

Second, Figure 3 shows the effect sizes Cohen's *d* for the pre-/post-change within each AVEM subscale or feature, based on the grouping of how teacher self-efficacy changed from pre- to post-intervention (δTSE; worsened: δTSE ≤ *M* – 1 SD; constant: *M* – 1 SD < δTSE < *M* + 1 SD; improved: δTSE ≥ *M* + 1 SD). Table 2 shows the means and confidence intervals for the AVEM subscales or features by group. In teachers whose *teacher self-efficacy worsened pre- and post-intervention* (*n* = 16), social support (SU) decreased by a medium effect size, *d* = −0.6, and experience of success at work (EB) and proactive problem-solving (OP) decreased by a small effect size (*d*_EB_ = −0.28, *d*_OP_ = −0.22). Distancing ability (DF) and willingness to work to exhaustion (VB) increased by small effect sizes (*d*_DF_ = 0.34, *d*_VB_ = 0.22). In teachers whose *self-efficacy remained constant* (*n* = 133), only distancing ability (DF) improved by a small effect size (*d*_DF_ = 0.2). In teachers whose teacher *self-efficacy improved pre- and post-intervention* (*n* = 22), distancing ability (DF) and general life satisfaction (LZ) increased by medium effect sizes (*d*_DF_ = 0.54, *d*_LZ_ = 0.79). Experience of social support (SU), experience of success at work (EE), inner calm and balance (IR), and proactive problem-solving (OP) increased by a small effect size each (*d*_SU_ = 0.22, *d*_EB_ = 0.34, *d*_IR_ = 0.35, *d*_OP_ = 0.49). Tendency for resignation in the face of failure (RT) and willingness to work to exhaustion (VB) shrunk by small effect sizes (*d*_RT_ = −0.34, *d*_VB_ = −0.27).

**Table 2:** Means, estimated confidence intervals, and effect sizes for each AVEM feature at both measurement points (baseline *t*_0_ and post-intervention *t*_1_), within the groups of change in teacher self-efficacy pre- and post-intervention (worsened, dTSE ≤ *M* – 1 SD; constant, *M* – 1 SD < dTSE < *M* + 1 SD; enhanced, dTSE ≥ *M* + 1 SD).

**Group pre-/post-change in teacher self-efficacy (dTSE)**		**Worsened (*****n*** **=** **16)**		**Constant (*****n*** **=** **133)**		**Enhanced (*****n*** **=** **22)**	
**AVEM feature**		***M* (95% CI)**	***d***	***M* (95% CI)**	***d***	***M* (95% CI)**	***d***
BA	*t*_0_	3.88 *(*2.96, 4.79*)*	0.17	4.09 *(*3.75, 4.43*)*	0.03	4.14 *(*3.13, 5.14*)*	0
	*t*_1_	4.19 *(*3.34, 5.03*)*		4.14 *(*3.84, 4.45*)*		4.14 *(*3.23, 5.04*)*	
BE	*t*_0_	4.06 *(*2.94, 5.19*)*	0.18	4.59 *(*4.21, 4.97*)*	0.09	4.95 *(*4.04, 5.87*)*	0.16
	*t*_1_	4.44 *(*3.46, 5.38*)*		4.78 *(*4.45, 5.11*)*		5.32 *(*4.37, 6.27*)*	
VB	*t*_0_	3.38 *(*2.56, 4.19*)*	**0.22**	4.89 *(*4.49, 5.28*)*	−0.17	5.73 *(*4.65, 6.8*)*	**−0.27**
	*t*_1_	3.75 *(*2.92, 4.58*)*		4.51 *(*4.15, 4.87*)*		5.05 *(*3.98, 6.11*)*	
PS	*t*_0_	3.69 *(*2.6, 4.77*)*	−0.03	4.26 *(*3.88, 4.64*)*	−0.11	4.91 *(*3.9, 5.92*)*	−0.07
	*t*_1_	3.63 *(*2.79, 4.46*)*		4.02 *(*3.66, 4.37*)*		4.73 *(*3.6, 5.85*)*	
DF	*t*_0_	5.06 *(*4.11, 6.02*)*	**0.34**	5.38 *(*5.05, 5.7*)*	**0.2**	5.32 *(*4.58, 6.05*)*	**0.54**
	*t*_1_	5.69 *(*4.82, 6.56*)*		5.77 *(*5.44, 6.09*)*		6.23 *(*5.56, 6.9*)*	
RT	*t*_0_	5.63 *(*4.37, 6.88*)*	0.17	5.45 *(*5.08, 5.82*)*	−0.12	5.45 *(*4.48, 6.43*)*	**−0.34**
	*t*_1_	6 (5.18, 6.82)		5.18 *(*4.81, 5.55*)*		4.68 *(*3.74, 5.62*)*	
OP	*t*_0_	3.44 *(*2.41, 4.46*)*	**−0.22**	4.11 *(*3.78, 4.43*)*	0.13	3.59 *(*2.94, 4.24*)*	**0.49**
	*t*_1_	3 (2.12, 3.88)		4.36 *(*4.01, 4.71*)*		4.41 *(*3.67, 5.15*)*	
IR	*t*_0_	5.63 *(*3.98, 6.14*)*	−0.17	4.8 *(*4.43, 5.17*)*	0.12	4.41 *(*3.48, 5.34*)*	**0.35**
	*t*_1_	4.69 *(*3.66, 5.71*)*		5.05 *(*4.72, 5.39*)*		5.14 *(*4.32, 5.95*)*	
EE	*t*_0_	4.38 *(*3.3, 5.45*)*	**−0.28**	4.93 *(*4.59, 5.28*)*	0.05	5.32 *(*4.54, 6.1*)*	**0.34**
	*t*_1_	3.69 *(*2.34, 5.04*)*		5.02 *(*4.70, 5.35*)*		5.95 *(*5.15, 6.75*)*	
LZ	*t*_0_	4.69 *(*3.5, 5.87*)*	0.13	4.81 *(*4.44, 5.18*)*	0.17	4.36 *(*3.47, 5.25*)*	**0.79**
	*t*_1_	5 (3.8, 6.2)		5.20 *(*4.81, 5.60*)*		6.14 *(*5.14, 7.13*)*	
SU	*t*_0_	5.75 *(*4.83, 6.67*)*	**−0.6**	4.45 *(*4.09, 4.81*)*	0.02	3.86 *(*3.25, 4.47*)*	**0.22**
	*t*_1_	4.56 *(*3.55, 5.57*)*		4.5 *(*4.12, 4.89*)*		4.23 *(*3.49, 4.97*)*	

*dTSE, pre-/post-change in teacher self-efficacy. AVEM features are given as stanine values (range = 1–9, M = 5, SD = 2). BA, subjective importance of work; BE, professional ambition; VB, willingness to work to exhaustion; PS, perfectionism; DF, distance ability; RT, tendency toward failure; OP, proactive problem-solving; IR, inner calm and balance; EE, experience of success at work; LZ, life satisfaction; SU, experience of social support; total N = 171; bold type values highlight medium effect sizes (Cohen's d, |0.2| = small effect, |0.5| = medium effect, |0.8| = large effect)*.

## Discussion

This study investigated, first, how teacher self-efficacy was related to general mental health and how it changed in the course of the preventive MBPGT (23). Furthermore, to better understand the relationship between teacher self-efficacy and mental health, we explored how teacher self-efficacy and its pre- and post-changes were related to work-related attitudes and behavior patterns.

The correlation between teachers' self-efficacy and their mental health status affirms teacher self-efficacy as a personal resource (37) along with its health benefits (38, 46, 47). Ancillary analyses revealed which work-related attitudes and behavior patterns were affected when teacher self-efficacy was high vs. when it was low. Differences among groups on the AVEM subscales were marked on those features that represent the resources psychological resistance and emotions: Regarding psychological resistance, teachers with (very) low self-efficacy have a lower distancing ability, lower proactive problem-solving, and lower inner calm and balance, as well as a higher tendency for resignation in the face of failure than teachers with (very) high teacher self-efficacy. On the component emotions, teachers with (very) low teacher self-efficacy reported a lower experience of success at work, lower life satisfaction, and lower social support than teachers with (very) high teacher self-efficacy. These results suggest how high teacher self-efficacy functions as a personal resource, namely by going hand in hand with high psychological resistance and overall positive emotions. If teacher self-efficacy is low, teachers are prone to experience higher levels of stress and seem to be unable to cope accordingly.

On the AVEM features that represent work commitment, teachers with low, medium, or high self-efficacy did not differ in their subjective importance of work, professional ambition, willingness to work to exhaustion, and striving for perfection. Although teacher self-efficacy should contribute to motivational processes through operative cognitions (38), teacher self-efficacy seems to matter less in performance-based motivation but more in the emotional attitude toward teaching, especially emotional stability and the experience of strain.

Teacher self-efficacy generally improved from pre- to post-intervention. Thereby, teachers whose teacher self-efficacy was the lowest at the beginning of the intervention showed the highest improvements in teacher self-efficacy. Additionally, participating teachers whose teacher self-efficacy was already high at the beginning of the intervention remained constant. The investigations using the AVEM features showed that as teacher self-efficacy improved, the teachers increased their distancing ability and their overall life satisfaction. Thus, teachers strengthened their ability to recover from work and gained confidence not only in topics related to teaching, but also in topics that indicate a stable and healthy personal background, like high psychological resistance factors and positive emotions. By contrast, teachers whose self-efficacy decreased post-intervention predominantly showed a reduction in experience of social support. The literature hails the experience of social support as an important, psychologically protective factor from adversity as well as an expression of well-being [cf. (60, 61)]. Skaalvik and Skaalvik (36) concluded that, within teaching as a profession, social support is a central resource that reduces negative influences of school-related stressors and the emotional response toward stress and directly enables teacher self-efficacy.

One could argue that the change in teacher self-efficacy resulted due to regression to the mean and, therefore, is estimated wrongly [cf. (62)]. In that case, it would have been more likely to observe that high teacher-self efficacy decreased pre- and post-intervention, instead of remaining constant. Moreover, the investigation in the AVEM subscales or features revealed that teachers whose self-efficacy improved post-intervention also improved resistance factors such as distancing ability. We have found earlier a similar improvement in resistance factors that went along with an improvement in mental health (30). Furthermore, the sample under investigation consisted of two independent subsamples from two consecutive school years. Identical analyses of the subsamples showed equal results as the joined sample. Thus, we conclude that regression to the mean did not relevantly influence our outcome.

Interestingly, teachers' self-efficacy increased regardless of how their general mental health status changed pre- and post-intervention. Although the MBPGPT is not designed to promote teacher self-efficacy specifically, it has shown to especially promote the experience of social support (60, in preparation), which is an important resource of self-efficacy in general (32). By focusing on promoting social support, the intervention may also indirectly promote self-efficacy. Moreover, the independence of teacher self-efficacy from changes in mental health can exclude cognitive biases of the intervention: For example, the interventions effect is not due to a “halo effect,” meaning that participants see improvements in every aspect. The independence could also indicate that once high teacher self-efficacy is achieved, it appears rather stable and may not be affected by taxing circumstances as easy as the current status of mental health. As teacher self-efficacy was already high at baseline, possibly, the respective cognitions influence how situations and experiences are appraised [cf. (63)]: Difficult situations may be rather evaluated as challenging than threatening. In this respect, mastering challenging situations may reinforce teacher self-efficacy and self-efficacy may stabilize over time. Therefore, it seems likely that if teacher self-efficacy is high, teachers can retain overall positive emotions and strong psychological resistance although their current mental health status may not be great.

Our results are based on a teachers' sample of the German state Baden-Wuerttemberg, which we characterize as a school system which is highly structured. State regulations may impose additional constraints on those teachers, besides classroom management and maintaining good personal relationships to students. So, it might be even more difficult for this sample to develop and maintain a high level of self-efficacy than other teachers from different school systems. Nevertheless, we think that our results can be generalized and we hypothesize the same linkage between self-efficacy and mental health for teachers in other school systems. It would be of interest to prove this hypothesis in a multicenter study.

### Limitations

First, the intervention was designed as a preventive measure: therefore, scientific research rather accompanied the integrated federal intervention than being its central focus. A control group was not planned in this context. So, the outcomes can only be interpreted cautiously as a result of the intervention. Moreover, the data reduction due to strict selection criteria from 742 registrations to 172 participants with both pre- and post-values was quite high. They likely resulted from the voluntary completion of the post-questionnaire, mistakes made within the personal code, or ending the intervention due to lack of time and change of address or workplace (28). Nevertheless, the conservative inclusion criteria were necessary to generate a valid pre-/post-sample. Despite the data reduction, the remaining sample was reliably high. Moreover, there were no significant differences between included and excluded participants regarding the key variables of interest, teacher self-efficacy, and mental health. The health benefits for true participants, as considered in the pre-/post-sample, have been proven (27, 28). Thus, although a control group was missing, it is likely that teacher self-efficacy improved through the intervention.

Second, the sample was possibly not representative for the general population of teachers, because only those who showed interest in the preventive measure could be assessed. The teachers who registered and participated at the intervention were probably in need of help and sought support. The interest in a preventive intervention may interfere with teacher self-efficacy: On the one hand, it is possible that their teacher self-efficacy was generally lower, yet norms for interpreting teacher self-efficacy scores are missing. On the other hand, the registration alone may have already stimulated the participants' teacher self-efficacy, because they took the first step for seeking help.

### Outlook and Practical Implications

For further research, it would be interesting to investigate if teacher self-efficacy and mental health change independently, as our results suggest. There may be variables which moderate or mediate the relationship and postpone a direct effect, such as dispositional differences, e.g., how intensely people generally perceive work-related stress. Focusing on cognitive influences on behavior such as the participants' attitude toward certain work-related objects [cf. (64)] may be a promising approach to better understand the psychological processes involved in changes in behavior and experience patterns which enhance mental health. Exploring the attitude–behavior link in the context of coaching and preventive interventions may lead to the identification of maladaptive attitudes. These could then be changed to adaptive attitudes through intervention, which consequently results in health-orientated and stress-reducing behavior [cf. (65)]. Perhaps, teacher self-efficacy—as an important construct within motivational processes (38)—can be identified as an adaptive attitude which fosters such behavior.

In addition, to further understand which dimensions of teacher self-efficacy are most closely related to mental health and change in preventive interventions, it would be interesting to examine specific dimensions of teacher self-efficacy by means of reliable subscales [e.g., Teacher Sense of Efficacy Scale (66, 67); Multidimensional Scale of Teacher Self-Efficacy, German: Multidimensionale Skala der Lehrer-Selbstwirksamkeit (68)]. Also, more specific measures for mental health such as physical parameters would be helpful in achieving more valid results than with subjective measures alone.

Of course, other aspects such as outcome dependent on school type or health care outcomes on students level would be also interesting and will be pursued in future research.

Despite the limitations mentioned above, important practical implications can be derived to encourage qualitative preventive measures. First, this study confirms teacher self-efficacy to be an important resource which correlates with good overall mental health and several work-related behavior and experience patterns, which mostly indicate strong psychological resistance abilities and general positivity. Therefore, fostering self-efficacy seems beneficial not only for teachers but likely also within other work-related contexts. Focusing on the client's work-related self-efficacy may be a promising and effective tool for coaches and counselors, especially when self-efficacy is distinctly low. By enhancing work-related self-efficacy, clients may strengthen their personal resources and improve their handling of work-related stress to prevent burnout in the long run.

Second, the AVEM features showed that, in order to establish and sustain a strong work-related self-efficacy, interventions should focus on promoting social support. This seems important in preventing a decrease in self-efficacy. Several studies support this conclusion by showing that social support and positive relationships constitute strong protective factors (19, 61, 69–73). Preventive interventions may also promote the client's ability to distance him- or herself from work-related content. This way teachers can regenerate, recover, and boost their overall life satisfaction. The next research steps include exploring the nature of the relation between teacher self-efficacy and mental health to identify any valid causal relation. In order to adapt preventive measures, it would be important to investigate to what extent preventive interventions should focus on teacher self-efficacy to foster health-related and work-related behavior patterns and emotions.

## Data Availability Statement

The datasets generated for this study are available on request to the corresponding author.

## Ethics Statement

The ethics committee of Albert-Ludwigs-University Freiburg has reviewed the submitted document and has no ethical or legal objections regarding the project and publications derived thereof.

## Author Contributions

MB, RP, and JB developed the treatment plan, research concept, and invited the participants. SM developed the scientific hypotheses and performed data analysis and drafted the initial version of the manuscript. MB, RP, AW, and CL directed the intervention and collected the data. RP and SM configured and matched the data. MB designed the figures. MB, RP, AG, and AW provided revisions and overall supervision. AG provided valuable improvements to the final draft of the manuscript. All authors approved the final version of the manuscript for submission.

## Conflict of Interest

The authors declare that the research was conducted in the absence of any commercial or financial relationships that could be construed as a potential conflict of interest.
